# The Efficacy of a Virtual Reality Exposure Therapy Treatment for Fear of Flying: A Retrospective Study

**DOI:** 10.3389/fpsyg.2021.641393

**Published:** 2021-06-15

**Authors:** Amihai Gottlieb, Glen M. Doniger, Yara Hussein, Shlomo Noy, Meir Plotnik

**Affiliations:** ^1^Center of Advanced Technologies in Rehabilitation, Sheba Medical Center, Ramat Gan, Israel; ^2^Department of Psychiatry, Sheba Medical Center, Ramat Gan, Israel; ^3^Ono Academic College, Kiryat-Ono, Israel; ^4^Department of Physiology and Pharmacology, Sackler School of Medicine, Tel Aviv University, Tel Aviv, Israel; ^5^Sagol School of Neuroscience, Tel Aviv University, Tel Aviv, Israel

**Keywords:** anxiety disorders, empirical supported treatments, computer/internet technology, behavior therapy, phobia/phobic disorders

## Abstract

**Background**: Fear of flying (FoF) is a phobia with 10–40% prevalence in the industrialized world. FoF is accompanied by severe economic, social, vocational, and emotional consequences. In recent years, virtual reality (VR)-based exposure therapy (VRET) for FoF has been introduced. Positive long-term efficacy of FoF-VRET has been reported by several studies, which, however, were limited by relatively small, non-representative samples and a lack of comparative pre/post functional efficacy outcome measures. Our objective was to evaluate the efficacy of a VRET treatment utilizing a large-scale VR system, experienced by a representative sample of self-referred individuals.

**Methods**: We conducted a retrospective survey. Of 274 individuals who received the treatment (over a period of 3 years), 209 met inclusion/criteria, and 98 agreed to participate. We mainly collected information regarding flight activity before and after treatment relying on evidence such as boarding passes and flight tickets. The primary outcome measures were (1) number of flights per month (FpM) and (2) number of flight hours per month (FHpM). For each participant, these outcomes were computed for the post-treatment period (≥6 months after FoF-VRET) and the corresponding pre-treatment period.

**Results**: FpM (mean ± SD) increased from 0.04 ± 0.06 to 0.16 ± 14 flights (*p* < 0.0001). FHpM rose from 0.19 ± 0.35 to 0.79 ± 0.87 h per month (*p* < 0.0001).

**Conclusion**: These results are indicative of FoF-VRET treatment efficacy. Future studies should evaluate long-term maintenance of the treatment effect and thus identify the optimal frequency for delivery of periodic booster treatments.

## Introduction

Airplanes are the safest, most common way to travel for long distance trips in the industrialized world (Transportation USDo, 2017; IATA, 2018). Fear of flying (FoF) is a common anxiety disorder in western countries, and its prevalence is estimated at 10–40% (Dean and Whitaker, 1982; Van and Diekstra, 2000; Oakes and Bor, 2010). Among those who suffer from FoF, 14% have never flown on an airplane, 6% have flown and say they will not fly again, and 10% have flown and say they will fly again only if there is no other choice (Ferrand et al., 2015). FoF may be secondary to phobias related to environmental conditions (e.g., altitude and severe weather) or situational phobias (e.g., claustrophobia) and may be comorbid to panic attacks and generalized anxiety disorder (Czerniak et al., 2016). Physiological and psychological anxiety symptoms of FoF may include panic attacks, fear, muscle tension, sweating, shortness of breath, heart palpitations, nausea, and dizziness (Kraaij et al., 2003). The costs of FoF for affected individuals, their families and society are substantial. FoF sufferers tend to avoid flying entirely, which may have serious social, vocational, and emotional consequences (Foreman et al., 2006). Societally, FoF results in significant cost to the airlines and incalculably reduced productivity and opportunity (Foreman et al., 2006).

Several pharmacological treatments exist for FoF including anti-anxiety medications like benzodiazepines (Greist and Greist, 1981). Other treatments are psychological interventions like exposure therapy (also called as systematic desensitization; Wiederhold et al., 2001). The most common treatment for FoF is cognitive behavioral therapy (CBT; da Costa et al., 2008), which focuses on creating neutral memories to replace the panic-inducing ones and may include relaxation techniques, psychoeducation, and exposure therapy. In exposure therapy, the FoF sufferer is exposed to the source of anxiety in a controlled manner, and this approach is considered as the most efficient treatment for FoF (Rothbaum et al., 2006; Rus-Calafell et al., 2013). Exposure therapy for FoF might involve simulating a flight or exposure to a stationary plane.

Over the last decade, virtual reality (VR) has become a viable method for administering exposure therapy for anxiety disorders. For example, several VR-based exposure treatments for post-traumatic stress disorder have been proposed (for review see Botella et al., 2015). As applied to FoF, virtual reality exposure therapy (VRET) involves creating a virtual airplane environment that simulates various aspects of flying using dynamic visual, auditory, and motion stimuli (Czerniak et al., 2016). Unlike exposure therapy using a real flight, this VR-based method allows the therapist to systematically control the level of the exposure intensity to a variety of elements (Schultheis and Rizzo, 2001). Notably, VRET for FoF (FoF-VRET) is most often implemented with a VR head mount display (e.g., Muhlberger et al., 2003; Tortella-Feliu et al., 2011) and thus lacks the ability to simulate motion. Large-scale VR systems that incorporate motion can be used to address this limitation and better simulate the flight experience.

There are few reports evaluating the clinical efficacy of FoF-VRET (e.g., Rothbaum et al., 2000, 2006; Maltby et al., 2002; Wiederhold et al., 2002; Muhlberger et al., 2003; Krijn et al., 2007; Tortella-Feliu et al., 2011). In a recent meta-analysis of 11 randomized trials, Cardoş et al. (2017) found FoF-VRET to be superior to control/standard FoF treatments. Only a few randomized trials have assessed efficacy in the months following treatment. For example, Rothbaum et al. (2000) reported the maintenance or enhancement of self-reported post-treatment improvements after 6 months for both VRET and standard exposure therapy groups. Further, at 6 months post-treatment, 79% of VRET participants and 69% of standard exposure therapy participants reported that they had flown (voluntarily) since completing the treatment. In another study, Muhlberger et al. (2003) found that 62% of VRET participants reported flying during the 6-month follow-up period. However, Maltby et al. (2002) reported that differences between VRET and an attention-placebo group observed immediately following treatment had disappeared after 6 months. In a randomized controlled trial, Rothbaum et al. (2002) found that 92% of VRET and 91% of standard exposure participants had flown 1-year post-treatment. Tortella-Feliu et al. (2011) found that 66% of VRET participants reported flying during the 1-year follow-up period. Finally, in a long-term follow-up study, Wiederhold and Wiederhold (2003) found that 85% of their 30 participants reported flying in the 3 years after completing several different VRET treatments.

Taken together, sample sizes in these studies were relatively small, and it is apparent that there is great variability (62–92%) in the prevalence of (voluntary) flying in the period following the conclusion of VRET treatment (Rothbaum et al., 2002; Muhlberger et al., 2003). Further, participants in such studies are not considered as representative of the general population as they have consented to an experimental treatment and are thus particularly motivated and amenable to the treatment. Most importantly, existing studies lack comparative pre/post functional efficacy outcome measures. To address these issues, better controlled studies with larger, more representative clinical samples are needed.

The research Center of Advanced Technologies in Rehabilitation (CATR) at Sheba Medical Center (Ramat Gan, Israel) has developed a FoF-VRET using a large-scale VR system (see Czerniak et al., 2016 for a full description of the setup; see also *Methods*). The FoF-VRET is provided as a personalized, flexible treatment; there are a number of variations that the therapist can apply to the treatment at his/her discretion in accord with professional experience. Until January 2019, more than 274 individuals have been treated.

The aim of the present study was to evaluate the efficacy of our FoF-VRET by retrospectively surveying individuals who received the treatment as a paid clinical service. Our primary objective was to evaluate whether flying habits changed after completion of the treatment.

## Materials and Methods

### Rationale

Between 2014 and 2018, 274 individuals were self-referred to receive FoF-VRET at the clinical virtual reality facilities in the virtual reality facilities of the rehabilitation hospital at Sheba Medical Center. We emailed 209 individuals who had completed the treatment and for whom we had an email address on file. In the email, we asked if they would be willing to participate in a phone survey regarding the FoF treatment they received (see section “Procedure” for more details). Among the benefits of this methodology are reduced bias associated with willingness to participate in experimental research, reduced bias associated with an onsite office interview by a clinician, and reduced “gratitude effect” consequent to pro bono research participation since participants in the present study paid out-of-pocket to obtain a clinical service.

### Participants

The Inclusion criteria were completion of the FoF-VRET treatment regimen at the clinical virtual reality facilities in the virtual reality facilities of the rehabilitation hospital at Sheba Medical Center and having an email address on file (to facilitate emailing of consent at initial contact). The Exclusion criteria were non-responsive to email, refusal to participate or <6 months after treatment completion.

Six months was set as the minimum time from treatment completion to allow a reasonable amount of time for participants to fly and for comparability to the literature (see section “Main Outcome Measures”). Of the 209 individuals we contacted, 98 actually participated. Individuals were excluded for the following reasons: 50 were non-responsive, 53 refused to participate, and eight were questioned <6 months from treatment completion. The majority of the participants were female (54%); mean age ± SD was 43.9 ± 13.3, and the range was 17–77 years. For technical reasons, age was not available for 15 participants, and gender was unavailable from one participant.

### Procedure

First, potential participants were emailed for their consent to participate; those who agreed were then contacted by phone to confirm their informed consent. Next, a structured phone interview was conducted. The interview consisted of three parts:

1. Confirmation of FoF-VRET treatment dates and recording the reason or reasons for self-referral.
2. Information on flight activity for the period following treatment completion and a corresponding period of identical length of time prior to treatment initiation. For each flight, participants reported their destination and flight duration. For verification purposes, participants were asked to furnish supporting material including boarding passes and passport stamps.
3. Questions about the FoF-VRET treatment experience, including whether they underwent other FoF treatments ±1 year before/after the FoF-VRET treatments.

### Main Outcome Measures

The primary measure of FoF-VRET efficacy was number of flights per month (FpM). The secondary outcome measure was number of flight hours per month (FHpM). For example, a participant interviewed 18 months after VRET completion reported the following flight information: to New York (11 h) in month +2, to London (5.5 h) in month +7, and to Eilat (1 h) in month +17. His/her outcome measures were thus FpM = (3/18) and FHpM = (17.5/18). Corresponding pre-treatment measures were calculated from data for the identical period of pre-treatment time.

### FoF-VRET Treatment

Refer to the Supplementary Material for a brief description of the FoF-VRET treatment (for a full description see Czerniak et al., 2016).

### Statistical Analyses

Non-parametric within-participant analyses (Wilcoxon signed-rank tests) were used to compare pre- and post-treatment FpM and FHpM levels as Kolmogorov-Smirnov tests indicated that these outcome measures do not follow a normal distribution (all *p* < 0.05). Alpha level was set at *p* < 0.05, two-tailed.

## Results

### Flight Activity Before and After FoF-VRET Treatment

**Figure 1:**
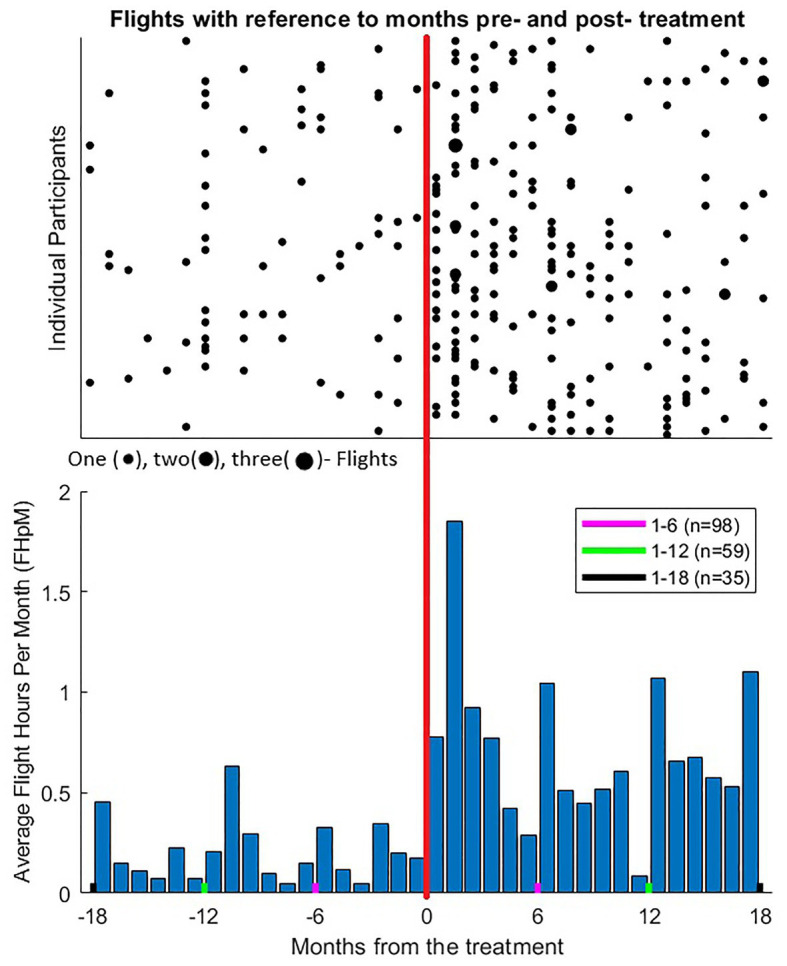
Flight activity 18 months before and after FoF-VRET treatment for individual participants. **(Top)** Each point represents at least one flight for the given month (see key below panel). Negative values on the abscissa reflect months pre-treatment, and positive values reflect months post-treatment, vertical orange line represents the month during which the treatment took place. Each horizontal row represents data from one participant. Data from 17 participants who did not fly before or after the treatment (i.e., reciprocal 18 months periods pre- and post-treatment) are not shown, yet these data were included in statistical analyses. Following treatment, mean ± SD FpM increased from 0.04 ± 0.06 to 0.16 ± 0.14 flights (*n* = 98; see also text for non-parametric comparisons). **(Bottom)** Mean flights hours per month (FHpM) across participants. Following treatment, mean FHpM raised from 0.19 ± 0.35 to 0.79 ± 0.87 h per month. Note that, for each participant, pre-treatment data were analyzed for the identical length of time as the post-treatment period at the time of data collection (see text). Thus, for all 98 participants, data were analyzed for 6 months pre/post treatment (red lines), for 64 participants data were analyzed for 12 months pre/post treatment (green lines), and for 35 participants data were analyzed for 18 months pre/post treatment (black lines). *Pre-hoc* analyses confirmed uniformity of distributions during overlapping periods for all three groups.

Participants showed a clear increase in flight activity post-treatment as compared to pre-treatment (Figure 1).

Regarding flight activity outcomes before and after treatment, within-participant analyses revealed a significant difference for FpM and FHpM before [FpM: median = 0, Interquartile Range (IQR) = 0.07, FHpM: median = 0, IQR = 0.28] and after (FpM: median = 0.13, IQR = 0.19, FHpM: median = 0.56, IQR = 1.05) treatment (Wilcoxon signed-rank tests, *Z* = 6.71, *p* < 0.0001, *Z* = 5.8, *p* < 0.0001, respectively).

**Figure 2:**
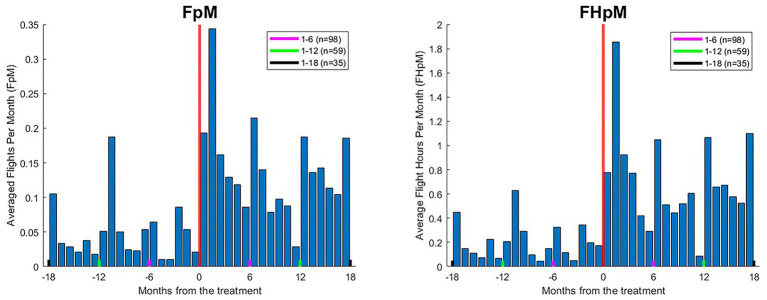
Mean flights per month (FpM; left) and flight hours per month (FHpM; right) across participants. Negative values on the *x*-axis reflect months pre-treatment, and positive values reflect months post-treatment. Following treatment, mean ± SD FpM increased from 0.04 ± 0.06 to 0.16 ± 0.14 flights; mean FHpM rose from 0.19 ± 0.35 to 0.79 ± 0.87 h per month. Note that, for each participant, pre-treatment data were analyzed for the identical length of time as the post-treatment period at the time of data collection (see the text). Thus, for all 98 participants, data were analyzed for 6 months pre/post treatment (red lines), for 64 participants data were analyzed for 12 months pre/post treatment (green lines), and for 35 participants data were analyzed for 18 months pre/post treatment (black lines).

Figure 2 shows FpM and FHpM across participants in the months before and after treatment.

### Reasons for Seeking FoF-VRET and Other FoF Treatments

**Figure 3:**
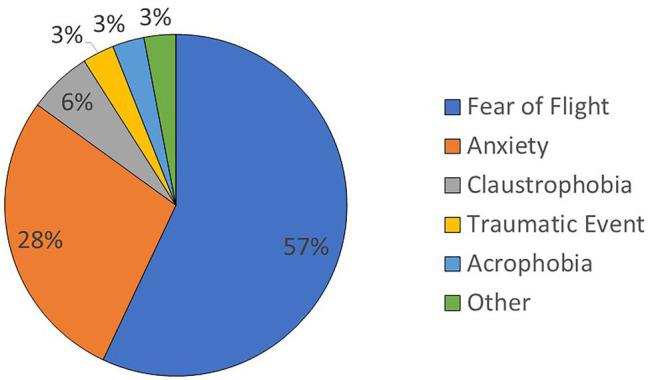
Distribution of reasons for seeking FoF-VRET treatment across participants. For participants who reported more than one reason, all the reported reasons are included.

To provide additional clinical background, Figure 3 shows the distribution of reasons for seeking FoF-VRET across participants, as reported during the phone interview.

**Figure 4:**
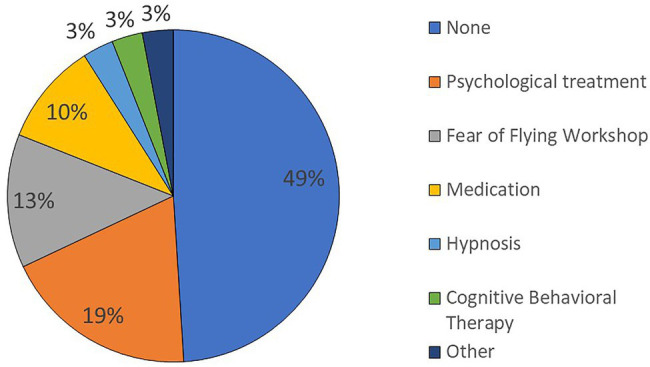
Distribution of other FoF treatments within 1 year of FoF-VRET. For participants who reported more than one treatment, all the reported treatments are included.

Figure 4 shows the distribution of other treatments for FoF within approx. ±1 year of FoF-VRET reported by participants. The distribution indicates that nearly half (48%) of participants did not engage in any other treatment. Among the other participants, psychological treatment (18%) and FoF workshops (12%) were most common; hypnosis (4%) and CBT (3%) were least common.

To confirm that our findings regarding FoF-VRET efficacy were not unduly affected by the additional treatments, we conducted a *post-hoc* analysis. Briefly, we split participants into “no other treatment” (49%) and “other treatment” groups. For each participant, we computed pre/post change (post-treatment minus pre-treatment; Δ) in FpM and FHpM, respectively. Man-Whitney tests revealed no significant differences between the groups (ΔFpM: *U* = 1237.0, *p* = 0.75, ΔFHpM: *U* = 1200.0, *p* = 0.95), suggesting that other treatments did not appreciably affect the increased flight activity we attribute to FoF-VRET.

## Discussion

This study aimed to determine the efficacy of FoF-VRET treatment using a retrospective follow-up questionnaire conducted over the phone. Our study is novel in that we evaluated individuals who voluntarily paid for and received treatment in our virtual reality center.

This FoF-VRET has several advantages over standard FoF exposure treatments. Firstly, it provides a safe, controlled environment that can be continuously monitored and manipulated by professional therapists and technicians. Secondly, it provides a highly detailed visual, auditory, and motion simulation of an actual flight experience rather than a static airplane. This provides better exposure to the fear-triggering factors, potentially inducing participant responses more similar to those elicited by real air travel. Finally, other measures like heart rate and blood pressure can be recorded during VR exposure therapy to provide therapists with more comprehensive clinical information.

### Efficacy

The current results show that number of flights and flight hours post-treatment significantly increased, reflecting treatment efficacy. These results are in line with other retrospective follow-up studies assessing the efficacy of other virtual reality-based FoF treatments (Rothbaum et al., 2000, 2002; Maltby et al., 2002; Muhlberger et al., 2003; Wiederhold and Wiederhold, 2003; Tortella-Feliu et al., 2011). The results of this study corroborate these prior studies and provide new evidence that those who benefited from the treatment continue to fly as long as 18 months after FoF-VRET treatment initiation.

While previous studies evaluating the efficacy of FoF-VRET used air travel in the post-treatment period (i.e., yes/no) as the sole (binary) outcome (e.g., Rothbaum et al., 2002; Wiederhold and Wiederhold, 2003), the current study introduces additional measures: flight frequency (i.e., number of flights per month) and flight hours per month in the post-treatment period. We believe that with the addition of these measures, we are able to provide the better evidence of treatment efficacy, as we show that treated participants not only fly more often, but also that they fly for longer durations. These results suggest that engaging in FoF-VRET leads participants to take flights they would not have been prepared to take prior to treatment.

In a recent meta-analysis of 11 randomized controlled trials, Cardoş et al. (2017) reported significant overall efficacy of a FoF-VRET intervention (*G* = 0.592) and a significant increase in flight activity at follow-up (*G* = 0.588), demonstrating the advantage of FoF-VRET treatment over control/traditional FoF treatments. However, their results also reveal the limitations of these trials due to poor study quality and small sample size. The authors suggest that reported effects may have been overestimated as a result of these issues. In contrast, our findings are based on a larger sample size and a more true-to-life (ecological) environment than those of the aforementioned studies.

### Other Results

Some of our results elucidate clinical aspects of FoF and its treatment. While most participants reported suffering specifically from FoF (acrophobia), a significant number of participants reported suffering from general anxiety. Furthermore, almost half (48%) of the individuals receiving FoF-VRET treatment reported that they did not engage in any other treatments at least 1 year prior to treatment, suggesting that half of those suffering from FoF are untreated and may avoid air travel.

### Limitations and Future Work

The current study is limited in several important ways. Firstly, to maximize sample size, we did not collect data at a fixed length of time from treatment (e.g., 1 year). Consequently, some adjustments to the data were required (e.g., standardizing the primary outcome measures to permit within-subject statistical comparisons). Secondly, the attrition rate was relatively high (51.8%), which may have affected the results. Although this level of attrition was higher than in other retrospective follow-up studies (13% in Rothbaum et al., 2002; 10% in Muhlberger et al., 2003; 10% in Wiederhold and Wiederhold, 2003; and 29.3% in Tortella-Feliu et al., 2011), a higher attrition rate may be expected for participants solicited to participate in a phone survey following receipt of a clinical treatment they paid for as compared to participants volunteering in research studies. A further limitation is that we interviewed many participants soon after treatment end and a smaller number of participants after an extended period post-treatment. Assuming attenuation of treatment effect with longer post-treatment duration, overall efficacy may thus have been inflated. However, in comparing FPM and FHPM for 0–9 and 9–18 months among the 22 participants with at least 1 flight in the 9 months post-treatment, we found no difference (see Supplementary Material). This issue should be further evaluated in a prospective, longitudinal study.

Finally, due to the nature of the study, only participants who actively sought the FoF-VRET were included. This sample of participants may have been biased as they likely had greater motivation to treat their FoF and fewer psychological barriers relative to others with fear of flying. Future studies may attempt to address this limitation by evaluating a broader sample.

As FoF severity was not assessed during treatment, future studies should examine the relation between FoF severity and FoF-VRET treatment outcome.

## Conclusion

Current results are indicative of FoF-VRET treatment efficacy. Air travel is an integral part of modern life in the industrialized world, and its prevalence is expected to grow as airfares continue to decrease and global economics entails more business travel (IATA, 2018). We can therefore expect a heightened awareness of FoF and an increase in referrals for suitable treatments including VRETs. Future studies should evaluate long-term maintenance of the treatment effect and consequently identify the ideal frequency for delivery of subsequent booster treatments.

## Data Availability Statement

Unidentified data supporting the conclusions of this article will be made available by the authors upon reasonable request.

## Ethics Statement

The studies involving human participants were reviewed and approved by the Sheba Medical Center Local IRB Committee. Written informed consent for participation was not required for this study in accordance with the national legislation and the institutional requirements.

## Author Contributions

AG performed some of the phone surveys, analyzed the data, and drafted the manuscript. GD drafted the manuscript. YH performed some of the phone surveys and analyzed the data. SN designed the study. MP designed the study, analyzed the data, and drafted the manuscript. All authors contributed to the article and approved the submitted version.

### Conflict of Interest

The authors declare that the research was conducted in the absence of any commercial or financial relationships that could be construed as a potential conflict of interest. Further, the authors declare no personal conflicts of interest. AG and MP are employees of Sheba Medical Center, and GD and YH were employed by Sheba Medical Center when conducting the research. Sheba Medical Center developed and offered the VR-based fear of flight treatment described here for clinical care of its patients.
